# Odontological Society of Pennsylvania

**Published:** 1892-07

**Authors:** 


### ODONTOLOGICAL SOCIETY OF PENNSYLVANIA.

The regular monthly meeting was held May 12, 1892. On the completion of the routine business the president introduced the essayist of the evening, Dr. Joseph Head, who read a paper entitled "Dental Litigation, Past and Present."

(For Dr. Head's paper, see page 510.)

### DISCUSSION.

*Dr. Boice.*—I would like to refer to one article Dr. Head mentioned. The Cummings patent was not for making rubber hard or rubber in plates or any shape or form, but simply for attaching porcelain teeth by means of metal pins into the rubber. The old Goodyear patent was for making plates of hard rubber, but the remarks on the injustice of the patent are undoubtedly true.

The Protective Association is the first association, I think, that has ever been organized for one object,—the protection of dentists. When it was first organized I joined it. It is necessary that we should be banded together in one association, as we have had nothing heretofore in dentistry, and I think it the duty of every member to talk to his neighbor and ask him to join. I asked Dr. Crouse some time ago how Pennsylvania was doing in the Protective Association. His language in reply was more expressive than elegant. Although we were one of the last States to see the importance of this work, can we not in the near future be one of the first in this good cause?

*Dr. Rehfuss.*—I am not aware that there are any law-points involved in the paper. It is an excellent history of dental patent litigations, which should have been its title, as there are so many different kinds of litigation in dentistry.

The Dental Protective Association has done wonderful work in protecting us. None but one who has gone through the whole patent matter, and who has thoroughly investigated nearly every case that has occurred in dental litigation, can realize the immense number pertaining to the dental profession that have come forward, particularly of late years. The history of the Goodyear patents is well known to you all, and have been very vividly and explicitly brought out by Dr. Head.

The previous speaker was in error when he said the Goodyear patent was not for vulcanizing rubber. It was for the process or composition, vulcanizing for *all purposes*. The Cummings patent was for the adaptation of vulcanized rubber to dental purposes. The reason for taking out the latter patent was, because the former, for the application of vulcanized rubber, would expire three or four years previous to that of the latter. Now we are threatened with a different class of dental patent litigations, and it seems to hinge entirely upon the bridge and crown patents. In the case of the International Tooth-Crown Company *vs.* Richmond, an entirely too broad construction was given by the judge, and it was erroneous, giving the patentee more than was claimed in the patent right. The patent gave him the right to cement bridges or continuous bands around the adjoining teeth, and thereby holding them in place permanently. The judge in this case gave it as his opinion that a crown was nothing more than a continuous band with a roof upon it, and allowed him a broad construction of his patent-claims, thereby making it illegal for any dentist to put in a bridge-piece which is fastened upon adjoining teeth by continuous bands or metal crowns.

The whole history of patent litigation has been a mistake throughout. A mistake in legal interpretations. The Dental Protective Association is doing a good work in proceeding against this, and in contesting these patents. As the majority of dental operation patents are worthless, I feel protected in doing any operation in dentistry, since I am a member of that Association. No man can afford the pecuniary means or time necessary to collect evidence in these cases or fight them single-handed. We must remember in our profession the old adage,—in union there is strength. Therefore I would call upon every member present who is not a member of the Association to join. There is but about one-fourth of the profession in that Association, and when we have the full number we can look forward to fighting not only unjust demands of patent agents, but other classes of abuses in dentistry of a legal nature.

(At this point Dr. Faught asked if it would not be well for those present not members of the Association to give their reasons for not being such. The answer in general was carelessness rather than a want of interest.)

*Dr. Bassett.*—This subject seems to admit of only a one-sided discussion, but I would like to narrate a little incident. I had the pleasure of joining this Association when I first heard of it, but the thing that influenced me was the experience of a classmate of mine. He had been practising in New York with some of these tooth-crown people, but finally separated from them and pursued some of the methods that they already had in their operations, but the Tooth-Crown Company soon followed him, and he undertook to fight them by employing a lawyer. He spent several hundred dollars, all the money he could spare, and finally had to give up the case because his money gave out, and he then took out a license from the company and went on. He was one and they were many, so they won the battle. As soon as this Protective Association was formed it seemed to me a very good way of combining and protecting ourselves at a very slight expense.

*Dr. Truman.*—I suppose many in the profession are like some present,—they have deferred attending to it. Those who have been sufferers from the old Goodyear Rubber Company do not require any urging to go into the Dental Protective Association. The trouble, the annoyance, the spy system that we all experienced at that time is well remembered, and we would be precisely in that same condition to-day were it not for Dr. Crouse and his Association.

I regard this Dental Protective Association as something more than a mere protection in a legal sense. There is nothing so powerful as a selfish interest to bind men together. As I said recently in an editorial on this very subject, that it is an important move for the man who is isolated and is yet able to feel through it that he is in touch with every other man in the profession. I feel more and more every day the necessity for men to get away from their petty, narrow jealousies. Dentists are full of it. We are broader in this country I think, taken as a whole, than anywhere else in the world. Yet there is room for improvement.

I remember when I first commenced to practise in my father's office, if a dentist came in he was considered an intruder, with the supposed intention of acquiring information without paying for it, and consequently the bars were put up in every office. This association will not only protect us legally, but will bring us together as we have never been before.

I hope those who are not members of the Protective Association will join. I know at times pecuniary difficulties are in the way, but Dr. Crouse makes it as easy as possible by the note plan. As has been said, he has expended time and money. I don't know another man so self-sacrificing. It requires just such a one to take the lead and stir the profession to its duty.

*Dr. Head.*—I think probably it might be well to explain to any dentist who might send their notes to Dr. Crouse that he will give them protection from any suits which may be entered against them from the time he receives such notes, but that he cannot let them legally join until the notes are paid. I know there has been a great deal of misunderstanding on that subject, many supposing that the notes meant that they were enrolled in the society, but Dr. Crouse is unable to do this for the reason that the money must be paid into the treasury absolutely before such enrolment can take place. To do that he would have to advance money himself, and, of course, where so many notes have been sent to him he cannot afford to do it.

Dr. Boice thought it appropriate that the Society should join as an individual to encourage the Association. A number of organizations have adopted this course.

Motion to that effect was made and carried.

Dr. Truman, under "Incidents of Practice," introduced a patient of Dr. Peeso with a full set of crown- and bridge-work which he thought worthy of examination.

Adjourned.

